# A review of epileptic seizure detection using machine learning classifiers

**DOI:** 10.1186/s40708-020-00105-1

**Published:** 2020-05-25

**Authors:** Mohammad Khubeb Siddiqui, Ruben Morales-Menendez, Xiaodi Huang, Nasir Hussain

**Affiliations:** 1 School of Engineering and Sciences, Tecnologico de Monterrey, Av. E. Garza Sada 2501, Monterrey, Nuevo Leon Mexico; 2 School of Computing and Mathematics, Charles Sturt University, 2640 Albury, NSW Australia; 3 College of Applied Studies and Community Service, King Saud University, Riyadh, Kingdom of Saudi Arabia

**Keywords:** Epilepsy, Applications of machine learning on epilepsy, Statistical features, Seizure detection, Seizure localization, Black-box and non-black-box classifiers, EEG signals

## Abstract

Epilepsy is a serious chronic neurological disorder, can be detected by analyzing the brain signals produced by brain neurons. Neurons are connected to each other in a complex way to communicate with human organs and generate signals. The monitoring of these brain signals is commonly done using Electroencephalogram (EEG) and Electrocorticography (ECoG) media. These signals are complex, noisy, non-linear, non-stationary and produce a high volume of data. Hence, the detection of seizures and discovery of the brain-related knowledge is a challenging task. Machine learning classifiers are able to classify EEG data and detect seizures along with revealing relevant sensible patterns without compromising performance. As such, various researchers have developed number of approaches to seizure detection using machine learning classifiers and statistical features. The main challenges are selecting appropriate classifiers and features. The aim of this paper is to present an overview of the wide varieties of these techniques over the last few years based on the taxonomy of statistical features and machine learning classifiers—‘black-box’ and ‘non-black-box’. The presented state-of-the-art methods and ideas will give a detailed understanding about seizure detection and classification, and research directions in the future.

## Introduction

The word epilepsy originates from the Latin and Greek word ‘epilepsia’ which means ‘seizure’ or ‘to seize upon’. It is a serious neurological disorder with unique characteristics, tending of recurrent seizures [1]. The context of epilepsy, found in the Babylonian text on medicine, was written over 3000 years ago [2, 3]. This disease is not limited to human beings, but extends to cover all species of mammals such as dogs, cats and rats. However, the word epilepsy does not give any types of clues about the cause or severity of the seizures; it is unremarkable and uniformly distributed around the world [1, 4].

Several theories about the cause are already available. The main cause is electrical activity disturbance inside a brain [1, 5, 6], which could be originated by several reasons [7] such as malformations, shortage of oxygen during childbirth, and low sugar level in blood [8, 9]. Globally, epilepsy affects approximately 50 million people, with 100 million being affected at least once in their lifetime [5, 10]. Overall, it accounts for 1% of the world’s burden of diseases, and the prevalence rate is reported at 0.5–1% [4, 11]. The main symptom of epilepsy is to experience more than one seizure by a patient. It causes a sudden breakdown or unusual activity in the brain that impulses an involuntary alteration in a patient’s behaviour, sensation, and loss of momentary consciousness. Typically, seizures last from seconds to a few minute(s), and can happen at any time without any aura. This leads to serious injuries including fractures, burns, and sometimes death [12].

### Seizure type

**Fig. 1:**
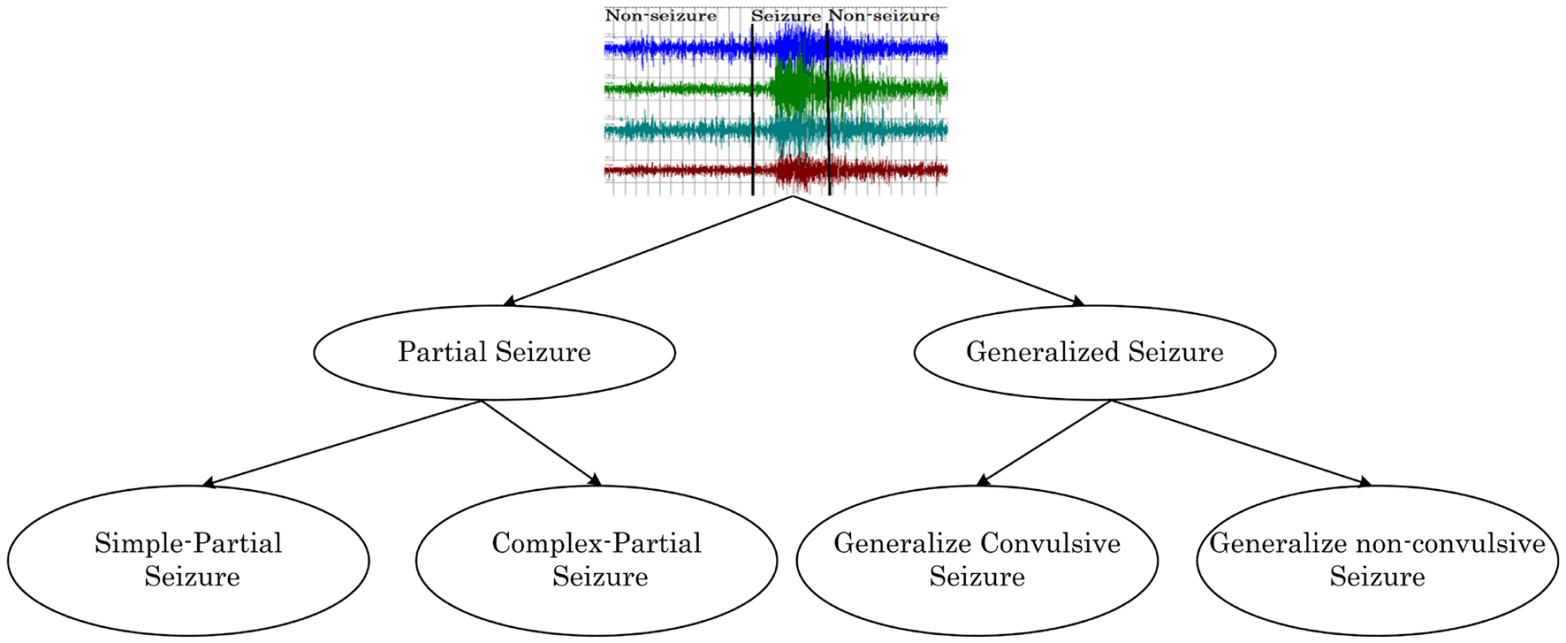
Types of seizure. Showing types of seizure and its sub-types

Based on the symptoms, seizures are categorized by neuro-experts into two main categories—partial and generalized [7, 13]—as shown in Fig. 1. Partial seizure, also called ‘focal seizure’, causes only a section of the cerebral hemisphere to be affected. There are two types of Partial seizure: simple-partial and complex-partial. In the simple-partial, a patient does not lose consciousness but cannot communicate properly. In the complex-partial, a person gets confused about the surroundings and starts behaving abnormally like chewing and mumbling; this is known as ‘focal impaired awareness seizure’. On the contrary, in the generalized seizures, all regions of the brain suffer and entire brain networks get affected quickly [14]. Generalized seizures are of many types, but they are broadly divided into two categories: convulsive and non-convulsive.

### Main contributions of the paper

In brief, the contributions of this paper are as follows:

1. We have done the review according to five main dimensions. First, researchers who adopted the EEG, ECoG or both for seizure detection; second, significant features; third, machine learning classifiers; fourth, the performance of the classifier during a seizure, and last, knowledge discovery (e.g., seizure localization).
2. Through study, it has been explored that an ensemble of decision trees (i.e., decision forest–random forest) classifier outperforms other classifiers (ANN, KNN, SVM, single Decision Tree).
3. We also suggest, how decision forest algorithms could be more effective for other knowledge discovery tasks besides seizure detection.
4. This study will help the researchers with their data science backgrounds to identify which statistical and machine learning classifiers are more relevant for further improvement to the existing methods for seizure detection.
5. The study will also help the readers for understanding about the publicly available epilepsy datasets.
6. In the end, we have provided our observations by the current review and suggestions for future research in this area.

The structure of the paper is organized as follows. “Role of data scientists in epileptic seizure detection” section gives the overview of machine learning experts in EEG datasets. The preliminaries requirements are provided in “A framework for seizure detection” section; it presents a general model of seizure detection and explains each step in a subsequent manner. “Publicly available datasets” section provides the details of benchmark datasets with their description. “Seizure detection based on statistical features and machine learning classifiers” section explains the review of literature work done on seizure detection using different machine learning classifiers, with a detailed comparison. “Seizure localization” section reviews the work done in identifying the affected lobes of the brain using machine learning classifiers. In “Problems identified in existing literature” section, we have explored the issues in the previous work and highlighted the gap. Overall, “observation about capable classifiers and statistical features” section reports our observations from the review about a suitable classifier and feature. “Research directions in seizure detection” section emphasizes the future directions in this research area, followed by “Conclusion” section on the summary of the paper.

## Role of data scientists in epileptic seizure detection

Applications of machine learning are significantly seen on health and biological data sets for better outcomes [15, 16]. Researchers/scientists on different areas, specifically, data mining and machine learning, are actively involved in proposing solutions for better seizure detection. Machine learning has been significantly applied to discover sensible and meaningful patterns from different domain datasets [17, 18]. It plays a significant and potential role in solving the problems of various disciplines like healthcare [17, 19–25]. Applications of machine learning can also be seen on brain datasets for seizure detection, epilepsy lateralization, differentiating seizure sates, and localization [26–29]. This has been done by various machine learning classifiers such as ANN, SVM, decision tree, decision forest, and random forest [26, 28].

Certainly, in the past, numerous reviews have been carried out on seizure detection along with applied features, classifiers, and claimed accuracy [27, 30–33] without focusing on the challenges faced by the data scientists whilst doing research on datasets of neurological disorders. Therefore, this article provides a detailed study of machine learning applications on epileptic seizure detection and other related knowledge discovery tasks. In this review, the collected articles are from well-known journals of their relevant field. These references are either indexed by *SCOPUS* or *Web of Science (WOS)*. Besides, we also considered some good ranked conference papers. Extensive literature is available covering the deep analysis of different features and classifiers applied on EEG datasets for seizure detection [31, 34, 35]. Both, feature extraction and applying classification techniques are challenging tasks. Previous literature reveals that for the past few years, interest has been increased in the application of machine learning classifiers for extracting meaningful patterns from EEG signals, which helps for detecting seizures, its location in the brain, and other impressive related knowledge discoveries [28, 36, 37]. Three decades ago, Jean Gotman [6, 38–40], analyzed and proposed the model for effective usage of EEG signals by applying different computational and statistical techniques for automatic seizure detection. Furthermore, the research has been carried out by different signal processing methods and data science methods to provide better outcomes [27, 34, 41–47].

## A framework for seizure detection

**Fig. 2:**
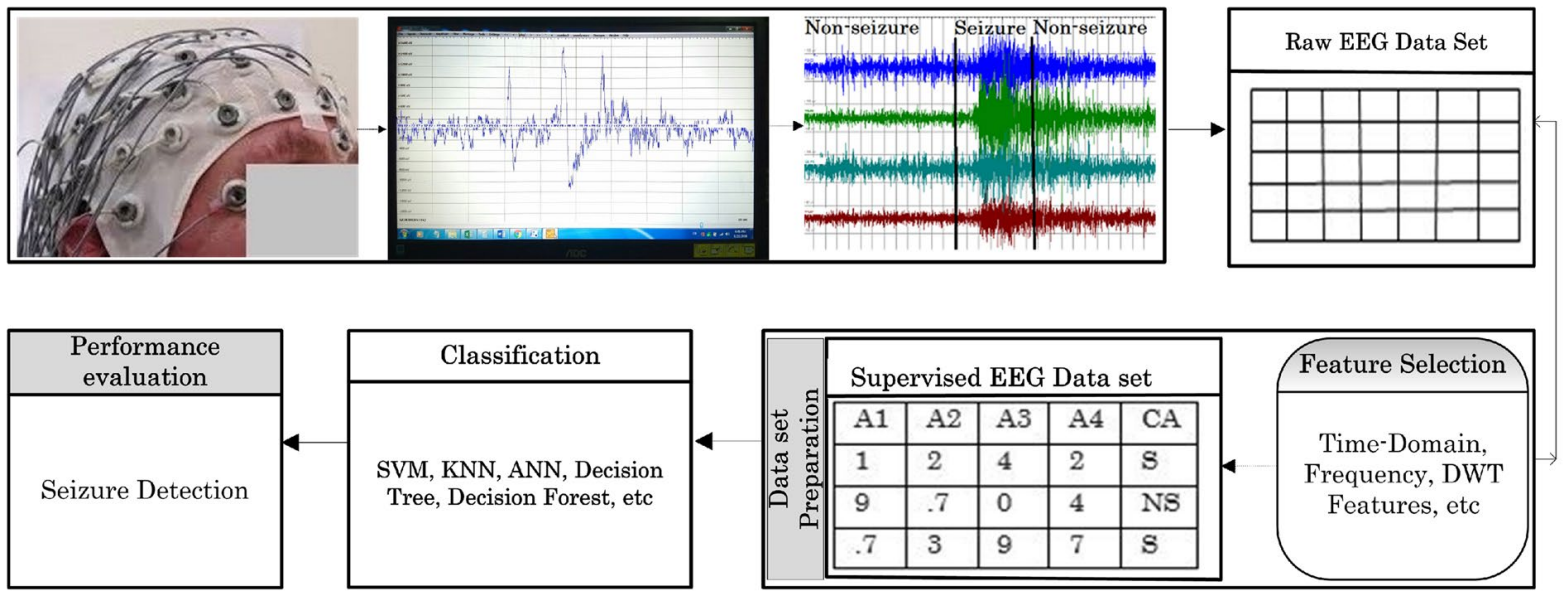
Basic model of epileptic seizure detection. This explains the basic steps to collect the dataset by EEG medium, display of raw EEG signals, transform EEG signals to two-dimensional table, feature selection, prepare the dataset with *seizure (S)* and *non-seizure (NS)*, apply machine learning classifier(s) and seizure detection, or other related tasks

In this section, we present a pictorial framework of the model used for seizure detection from an EEG/ECoG seizure dataset, illustrated in Fig. 2. The process comprises four steps: Data Collection, Data Preparation, Applying Machine Learning Classifiers and Performance Evaluation.

### Data collection

The initial requirement is to collect the dataset of brain signals. For this, different monitoring tools are used. Typically, the mostly used devices are EEG and ECoG, because their channels or electrodes are implanted by glue on the surface of the scalp as per 10–20 International system [48] at different lobes. Each of them has a wire connection to the EEG device, providing timely information about the variations in voltage, along with temporal and spatial information [49]. As highlighted in Fig. 2, the EEG channels are placed on the subject’s scalp, and the electrical signals are read by the EEG monitoring tool and it displays these raw signals over the screen. Further, these raw signals have been carefully monitored by the analyst and classified into ‘seizure’ and ‘non-seizure’ states.

### Data transformation

After data collection, the next crucial step is to transform the signal data into a 2-D Table format. The reason for this is to make it easier for analysis and provide necessary knowledge like seizure detection. This datum is raw because it has not been processed yet. Therefore, it will not be suitable to give relevant information. To do the processing, different feature selection modalities have been applied. This step also presents the dataset as supervised, which means that it provides the class attribute with possible class-values.

### Dataset preparation

For data transformation, data processing is a decisive step to extract meaningful information from the collected raw dataset. As such, different feature extraction techniques have been used; as shown in Table 1. These methods are generally applied to the extracted EEG signal dataset [31, 34]. The raw dataset becomes rich in terms of different statistical measure values.

**Table 1:** Feature extraction methods and features used on EEG signal dataset

Feature extraction methods	Relevant features
Time-domain features	Mean, variance, mode, median, skewness, kurtosis, max, min, zero crossing, line length, energy, power, Shannon entropy, sample entropy, approximate, entropy, fuzzy entropy, hurst exponent, standard deviation
Frequency-domain features	Spectral power, spectral entropy, energy, peak frequency, median frequency
Time–frequency-domain features	Line length, min, max, Shannon entropy, approximate entropy, standard deviation, energy, median, root mean square
Discrete Wavelet Transformation (DWT)	Bounded variation, coefficients, energy, entropy, relative bounded, variation, relative power, relative scale energy, variance, standard deviation
Continuous Wavelet Transformation (CWT)	Energy’s standard deviation, energy, coefficient z-score, entropy,
Fourier Transformation (FT)	Median frequency, power, peak frequency, spectral entropy power, spectral edge frequency, total spectral power

After feature extraction processing, the dataset becomes more informative that it ultimately helps the classifier for retrieving better knowledge.

### Applying machine learning classifiers and performance evaluation

To achieve a high accuracy of seizure detection rate and explore relevant knowledge from the EEG processed dataset, different supervised and unsupervised machine learning have been used.

#### Classification

In classification, a dataset *D* has a set of ‘non-class attributes’, and a ‘class attribute’. They are the principal components and their pertinent knowledge is very important, as both have a strong association for potential classification. The target attribute is defined as the ‘class attribute’ *C*, and it comprises more than one class values, e.g., *seizure* and *non-seizure*. On the contrary, attributes $A=\{A_1,A_2.A_3 \ldots A_n\}$ are known as ‘non-class attributes’ or predictors [50, 51]. The following classifiers have been popularly used in seizure detection. Common classifiers such as SVM [52], decision tree [53] and decision forest [54] are applied to the processed EEG dataset for seizure detection.

#### Performance evaluation

The accuracy of the obtained results is used to evaluate different methods. The most popular training approach is tenfold cross-validation [55], where each fold, i.e., one horizontal segment of the dataset is considered to be the testing dataset and the remaining nine segments are used as the training dataset [56, 57].

Except for the accuracy, the performance of the classifiers is commonly measured by the following metrics such as precision, recall, and f-measure [58]. These are based on four possible classification outcomes—True-Positive (TP), True-Negative (TN), False-Positive (FP), and False-Negative (FN) as presented in Table 2.

**Table 2:** Classification outcomes

Acronym	Detection type	Real-world scenario
TP	True-positive	If a person suffers to ‘seizure’ and also correctly detected as a ‘seizure’
TN	True-negative	The person is actually normal and the classifier also detected as a ‘non-seizure’
FP	False-positive	Incorrect detection, when the classifier detects the normal patient as a ‘seizure’ case
FN	False-negative	Incorrect detection, when the classifier detects the person with ‘seizure(s)’ as a normal person. This is a severe problem in health informatics research

This table describes each parameter metric considering seizure and non-seizure case

Precision is the ratio of true-positives to the total number of cases that are detected as positive (TP+FP). It is the percentage of selected cases that are correct, as shown in Eq. 1. High precision means the low false-positive rate.

1 $$\begin{aligned} \text{Precision}=\frac{TP}{TP+FP}\times 100\% \end{aligned}$$

Recall is the ratio of true-positive cases to the cases that are actually positive. Equation 2 shows the percentage of corrected cases that are selected.

2 $$\begin{aligned} \text{Recall}=\frac{TP}{TP+FN}\times 100\% \end{aligned}$$

Despite getting the high Recall results of the classifier, it does not indicate that the classifier performs well in terms of precision. As a result, it is mandatory to calculate the weighted harmonic mean of Precision and Recall; this measure is known as F-measure score, shown in Eq. 3. The false-positives and the false-negatives are taken into account. Generally, it is more useful than accuracy, especially when the dataset is imbalanced.

3 $${\text{F-measure}} = 2 \times \frac{{({\text{Precision}} \cdot {\text{Recall}})}}{{{\text{Precision}} + {\text{Recall}}}}$$

## Publicly available datasets

For data scientists and researchers, a dataset used is important for evaluating the performance of their proposed models. Similarly, in epileptic seizure detection, we need to capture the brain signals. EEG recording is the most used method for monitoring brain activity. These recordings play a vital role in machine learning classifiers to explore the novel methods for seizure detection in different ways such as onset seizure detection, quick seizure detection, patient seizure detection, and seizure localization. The significance of publicly available datasets is that they provide a benchmark to analyze and compare the results to others. In the following section, we will describe the popular datasets that are widely used on epilepsy.

### Children Hospital Boston, Massachusetts Institute of Technology—EEG dataset

This dataset is publicly available on a physionet server and prepared at Children Hospital Boston, Massachusetts Institute of Technology (CHB-MIT) [59, 60]. It can be collected easily via Cygwin tool which interacts with the physionet server. It contains the number of seizure and non-seizure EEG recordings for each patient of the CHB [61]. The dataset comprises 23 patients; 5 males, aged 3–22 years, and 17 females aged 1.5–19. Each patient contains multiple seizure and non-seizure recording files in European data format (.edf), representing the spikes with seizure start and end time, which is easily visible at a browser called an ‘EDFbrowser’. The primary datasets are in the 1-D format, containing EEG signals that are obtained through the different types of channels that were placed on the surface of the brain as per 10-20 International System. All these signals of the dataset were sampled at the frequency of 256Hz.

### ECoG Dataset, Epilepsy Centre, University of California

This is a publicly available dataset of electrocorticogram (ECoG) signals from an epileptic patient, which was collected from the Epilepsy Center, University of California, San Francisco (UCSF) [62]. It was originally collected by implanting 76 electrodes on the scalp in both invasive (12-electrodes) and non-invasive manner (64-electrodes). It comprises 16 files altogether. Out of these, eight files ($F1, F2, \cdots F8$) are classified as ‘pre-ictal’ meaning the stage before the seizure. The rest of the files ($F9, F10, F11, \cdots F16$) represent the ‘ictal’ stage data. The collected data are sampled at the frequency of 400 Hz (i.e., 400 cycles/s) and the total duration is 10 s. As a result, there are (400 cycles/s $\times$10 s) 4000 cycles in each file [63].

### The Freiburg—EEG dataset

This dataset was collected from the invasive EEG recordings of 21 patients (8 males aged 13–47 years, 13 females aged 10–50 years) suffering from medically intractable focal epilepsy. It was recorded during an invasive pre-surgical epilepsy monitoring at the Epilepsy Centre of the University Hospital of Freiburg, Germany [64]. Out of 21 patients, 13 patients had 24 h of recordings, and 8 patients had less than 24 h. These recordings are inter-ictal, and together they provide 88 seizures.

### Bonn University—EEG dataset

The dataset comprises five subsets, where each one denoted as (A–E) contains 100 single-channels recording, and each of them has a 23.6 s duration, captured by the international 10–20 electrode placement scheme. All the signals are recorded with the same 128-channel amplifier system channel [65].

### BERN-BARCELONA—EEG dataset

This dataset comprised EEG recordings derived from five pharmacoresistant temporal lobe epilepsy patients with 3750 focal and 3750 non-focal bivariate EEG files. Three patients were seizure-free, with two patients only having auras but no other seizures following surgery. The multichannel EEG signals were recorded with an intracranial strip and depth electrodes. The 10–20 positioning was used for the electrodes’ implantation. EEG signals were either sampled at 512 or 1024 Hz, depending on whether they were recorded with more or less than 64 channels. According to the intracranial EEG recordings, they were able to localize the brain areas where seizures started for all five patients [66]. This dataset is good for the seizure localization purpose.

## Seizure detection based on statistical features and machine learning classifiers

This section explains the comprehensive detail of work on seizure detection using statistical features, classifiers—‘black-box’ and ‘non-black-box’. They are illustrated in Table 3. In brief, the ‘black-box’ classifiers are those which provide the accuracy without mentioning the reasons behind the results such as ANN and SVM [67]. They are unable to explain their classification steps. Whereas, ‘non-black-box’ classifiers such as decision forest and random forest can able to explain each step of the processing, which is human-understandable. As a result, it helps in human-interpretable knowledge with high accuracy [68].

**Table 3:** Overview of existing work on seizure detection using—machine learning classifiers, features, performance score, performance metrics, datasets, and Authors

Classifier(s)	Feature(s)	Performance (%)	Performance metrics	Dataset	Authors
SVM	Vector	96	Sensitivity (Sen)	CHB-MIT	Shoeb and Guttag [41]
Random forest	Time and frequency	93.8	Senstivity	EPILEPSIAE	Donos et al. [44]
ANN	Line length	99.6	Classification accuracy (Class Acc)	BONN	Guo et al. [69]
Burst detection algo	Line length	84.27, 84,85.7	Acc, Sen, Specificity (Spec)	NICU, Belgium	Koolen et al. [70]
Normalization	Line length	52	ROC	CHB-MIT	Logesparan et al. [71]
ELM and BPNN	SE	95.6	Class Accuracy	BONN	Song and Lio [72]
SVM and ELM	AE and SE	95.58	Class Accuracy	BCI Lab, Colarodo	Zhang et al. [73]
SVM	DWT	94.8	Avg Accuracy	CHB-MIT	Ahmad et al. [74]
GMM	Spectral, hybrid, temporal	87.58	Avg Accuracy	CHB-MIT	Gill et al. [75]
Random forest	PCA, STF, Moving Max	97.12, 99.29, 0.77/h	Sen, Spec, FPR	CHB-MIT	Orellana and Cerqueira [76]
Random forest and k-NN	Spectral power	80.87, 47.45, 2.5/h, 56.23	Sen, Prec, FPR, F-meas	CHB-MIT	Birjandtalab et al. [77]
Boosting	Stockwell	94.26, 96.34	Sen, Spec	Freiburg	Yan et al. [78]
SVM, MLP, KNN, Naïve bayes	Energy	98.75	Class Acc	EPILEPSIAE	Amin et al. [79]
Random forest	Entropy and DWT	98.45	Class Acc	BONN	Mursalin et al. [80]
SVM	Time–Frequency	90.62, 99.32	Sen, Spec	CHB-MIT	Zabihi et al. [81]
Random forest	Time-domain	96.94	ROC curve	Kaggle	Truong et al. [82]
SVM, LDA, QDA, LC,PC, DT, KNN, UDC, PARZEN	Time–frequency	84, 85	Sen, Spec	CHB-MIT	Fergus et al. [83]
SVM	DWT	86.83	Confusion Matrix	CHB-MIT	Chen et al. [84]
SVM and neural network	DWT and CWT	99.1	Overall Acc	BONN	Satapathy et al. [85]
ELM	Time–frequency	97.73, 0.37/h	Sen, false alarm rate	Freiburg	Yuan et al. [86]
SVM	DWT	99.38	Class Acc	BONN	Subasi et al. [87]
LS-SVM	FFT and DWT	100	Class Acc	BONN	Al Ghayab et al. [88]
SVM and Naïve bayes	Entropy, RMS, variance, energy	96.55, 95.63, 95.7	Sen, Spec, Acc	CHB-MIT	Selvakumari et al. [89]
LS-SVM	8 types of Entropies	100, 99.4, 99.5	Sen, Spec, Acc	BONN	Chen S et al. [90]
ANN	Spectral power	86	F-meas	CHB-MIT	Birjandtalab et al. [91]
KNN and GHE	-	100	Class Acc	BONN	Lahmiri and shumel [92]
Random forest	DWT	99.74, 0.21/h	Sen, FPR	BONN and Freiburg	Tzimourta et al. [93]
Random forest	STFT, mean, energy and std dev	96.7	Class Acc	BONN	Wang et al. [94]
Random forest, SVM, KNN, and Adaboost	28 statistical and time–frequency features	97.6, 94.4, 96.1, 92.9, 98.8, 0.96	Sen, Spec, Acc, PPR, NPR, ROC	Bern-Barcelona	Raghu and Sriraam [95]
ANN,KNN,SVM, and Random forest	Mean, std dev, power, skewness, kurtosis, absolute mean	100	Overall Accuracy	Freiburg and CHB-MIT	Alickovic et al. [96]
SVM	Energy	99.5	Class Acc	BONN and Barcelona	Fasil and Rajesh [97]
SVM and Random forest	10-time and frequency	0.98	ROC(AUC)	EPILEPSIAE	Manzouri et al. [98]
LS-SVM	DCT, SVD, IMF, DCT-DWT,	91.36	Acc, Sen, Spec	Freiburg	Parvez and Paul [99]
SysFor and Forest CERN	9 statistical features	100	Class Acc	Epilepsy Centre UCSF	Siddiqui et al. [63]
Random forest	L1-penalized robust regression (L1PRR)	100	Class Acc	BONN	Hussein et al. [100]
SVM, NB, KNN, random forest, logistic model Trees (LMT)	15-features	97.40, 97.40,97.50	Acc, Sen, Spec	BONN	Mursalin et al. [101]
Random forest	IMF	98.4,98.6,96.4	Sen, Spec, Acc	BONN	Sharma et al. [102]
ANN	Time–frequency	100	Overall Acc	BONN	Tzallas et al. [103]
Decision forest–Random forest, Boosting	9 statistical features	96.67,74.36, 84.06	Pre, Rec, F-measure	CHB-MIT	Siddiqui et al. [104]

### Seizure detection based on statistical features

If we apply machine learning classifier(s) directly to raw EEG/ECoG datasets, it may not produce enough sensible patterns. Therefore, selecting significant and capable statistical features from EEG and ECoG raw datasets is one of the challenges and a crucial task. The nature of EEG and ECoG signals is very complex, non-stationary and time-dependent [105–107]. As such, we can apply the machine learning classifier(s) to the processed datasets, which will ultimately assist to solve various neurological problems; for example, identifying seizure’s stages, accurate seizure detection, fast detection, etc. In Table 3, we summarize a review of several studies.

The significant statistical features were extracted by different types of transformation techniques; discrete wavelet transformations (DWT), continuous wavelet transformation (CWT), Fourier transformation (FT), discrete cosine transformation (DCT), singular value decomposition (SVD), intrinsic mode function (IMF), and time–frequency domain from EEG datasets [34, 71, 79, 108]. Logesparan et al. [34] used different types of feature extraction methods for seizure detection, but they reported that two features—‘line length’ and ‘relative power’—are the good performers for seizure detection. Guerrero-Mosquera [109] applied three time-domain features—line length, frequency, and energy on the raw EEG dataset. These features claim to be suitable for seizure detection and other brain-related applications such as computer interface (BCI). The claimed performance was evaluated using the following metrics such as sensitivity, specificity, F-score, receiver operating characteristics (ROC) curve, and percentile bootstrap measures. Duo Chen [84] used DWT with the SVM classifier on two benchmark datasets—CHB-MIT and Bonn University, achieved seizure detection accuracies of 92.30% and 99.33%, respectively. Ramy Hussein et al. [100] proposed a new featured L1-penalized robust regression (L1PRR) for seizure detection, the issue with their approach is computational complexity. Zavid and Paul [99] focused on classifying the ‘ictal’ and ‘inter-ictal’ states, where they used four features DCT, DCT-DWT, SVD, and IMF; the obtained signals are further classified by LS-SVM due to less computational cost.

Several researchers have contributed to seizure detection using a single feature [108, 110]. The feature ‘line length’ [108, 110] was applied to an EEG dataset; approximately 4.1 s of mean detection latency is recorded at a false alarm rate of 0.051 Fp/h. Further, Guo et al. [69] also used ‘line length’ but with the ANN for classifying the records obtained by EEG signals. Their automated seizure detection accuracy is 99.6%. A system was proposed by Koolen et al. [70] to detect seizures from EEG recordings. This detection system uses a single feature—‘line length’. The performance of this system shows 84.27% accuracy, 84.00% sensitivity and 85.70% specificity, which are comparatively lower than the results of Guo et al. [69].

After 3 years of study on several of statistical features [34], Logesparan et al. [71] proposed the ‘line length’ feature for normalization and discrimination of class values from EEG datasets. It is noted that ‘line length’ could be taken as the strongest feature and provides considerable output. Based on previous studies, the ‘line length’ can be taken with other features, and the result would be more promising, specifically in machine learning. This is because the dataset dimension would also increase with meaningful statistical information in the attributes.

Some other studies on seizure detection based on a single feature, i.e., entropy and its sub-types such as approximate entropy (AE) and sample entropy (SE), have also been done [45, 72, 73, 111]. The entropy feature helps to find the random behaviour of EEG signals and takes depth benefits in measuring the impurity of the signals [112, 113]. The entropy feature has been used widely where data are in the form of signals such as ECG, [114], EEG, and ECoG [36]. This helps in further steps of the detection model.

Acharya et al. [111] used four different types of entropy-based features: sample entropy, approximate entropy, phase entropy (S1), and phase entropy (S2) of the EEG datasets. The processed dataset from these entropy features was used for seizure detection. In another study, Chen et al. [90] used eight different kinds of entropy feature—approximate, sample, spectral, fuzzy, permutation, Shannon, conditional and correction conditional on a raw EEG dataset; further, the processed data were classified into three class values: ‘ictal’, ‘inter-ictal’ and ‘normal stage’, and their accuracy is 99.50%. A tool was proposed by Selvakumari et al. [89] using four features—entropy, root mean square (RMS), variance, and energy. Based on these features, the detection was done using SVM and naïve Bayesian classifiers with a reported accuracy of 95.63%. The tool is also able to find the seizure region in the brain; however, they did not mention the exact percentage of seizure location. Song and Li [72] built classification models by two classifiers—Extreme Learner Machine (ELM) and the back-propagation neural network (BPNN). Overall, their findings show 95.6% of classification accuracy with less execution time. Yong Zhang et al. [73] applied two entropy features—AE and SE on two different classifiers—ELM and SVM for processing EEG dataset. The SE features with ELM provide good classification accuracy compared to the AE feature whilst detecting the seizure.

The energy feature has been significantly used in seizure detection [115]. It plays a vital role particularly when the seizure is detected by the epoch- or windows-based method. This means that the EEG signals are divided into various segments [79, 94]. An exponential energy feature has been introduced by Fasil and Rajesh [97], which helps in identifying the irregularities in amplitude EEG signals.

*Observations* This section has provided an overview of the contributions of statistical features to seizure detection and their importance. Some researchers detect seizures using multiple sets of features, whilst others select a single feature such as ‘line length’. We recommend the ‘line length’ feature to be in the list of the set of suitable features for seizure detection because it is helpful in measuring the EEG signals complexity. It plays a sensitive role in the changes at the frequency and amplitude of signals. As a result, it helps to discriminate against the ‘seizure’ and ‘non-seizure’ cases. However, from the data science point of view, it is very important to see the various perspectives of each brain signals by observing other statistical features. Furthermore, we also suggest not to use the irrelevant feature(s) as they will unnecessarily increase the dataset size which results in an increase in computational time and gives insensible patterns too. As a result, it becomes a hassle to machine learning classifiers and users rather than providing the benefit. Some researchers [95, 98, 101] used a large number of features, which increases the attribute size, and results in more computational time and less accuracy. So, if we take the fewer features as previous researchers have done [71, 73, 79] this will give the low-dimensional dataset, which will not be fruitful for the knowledge discovery process. The next section illustrates the seizure detection by ‘black-box’ classifiers. As far as the classification purpose is concerned, it would be better to take more relevant statistical features, which can be integrated into knowledge discovery and a good performance rate.

### Seizure detection based on black-box classifiers

The classifiers such as SVM, ANN, and KNN are considered as prominent ones due to their remarkable performances in different domains [67, 116]. Each technique has its pros and cons, and ‘black-box’ methods are not an exception to this [104]. Even though these classifiers contribute well to brain datasets, some of the relevant works on seizure detection using these classifiers are reported here.

The study of Satapathy et al. [85] was based on two ‘black-box’ approaches—SVM and Neural networks using different kernel methods for seizure detection against a large EEG dataset. The performance of each classifier is measured independently by the majority voting system, and it was found that SVM was more capable than other neural networks. Subasi et al. [87] proposed the solution to detect seizure using a hybrid approach of SVM, genetic algorithm (GA), and particle swarm optimization (PSO). The method achieved impressive accuracy, i.e., 99.38%, but the problem is that the classifier trains the dataset twice, one for SVM-GA and another for SVM-PSO. This could be a time-consuming.

Shoeb and Guttag [41] performed seizure detection on their arranged dataset of Child Hospital Bostan, MIT (CHB-MIT) [60] using SVM with the vector feature and achieved the estimated accuracy of 96%. Dorai and Ponnambalam [42] came with an idea of the epoch, which means dividing the dataset into smaller time frames. Further, they applied an ensemble of four ‘black-box’ approaches—LDA, KNN, CVE, and SVM on these epoch EEG datasets. This approach provides the prediction of onset seizures 65 s earlier. Classifying the EEG data into two class ‘ ‘seizure” and ‘non-seizure’, Birjandtalab et al. [117] used a Gaussian mixture model (GMM) before detecting the seizure, and obtained 90% accuracy with 85.1% F-measure. They also raised the issue of class imbalance in their dataset. Tzallas et al. [103] used time–frequency-domain features with ANN for the EEG dataset and obtained 100% accuracy for the ‘seizure’ and ‘non-seizure’ classification problem; with epochs’ datasets the accuracy is 97.7% from (A, B, C, and D) for ‘non-seizure’ and set E for ‘seizure’ epoch classes. Amin et al. [79] extracted relative energy features from the DWT method, and four classifiers—SVM, MLP, KNN, and Naïve Bayes—were applied for the classification purpose, the result shows 98% of SVM accuracy, which outperforms remaining classifiers. A framework had been proposed by K. Abualsaud et al. [118] using the ensemble of ‘black-box’ classifiers for automated seizure detection on noisy EEG signals, and the reported classification accuracy is 95%. However, the ensemble approach did not provide good accuracy as desired because all four classifiers were ‘black-box’.

In 2018, Lahmiri et al. [92] used generalized Hurst exponent (GHE) and KNN, to propose a system for identifying the ‘seizure’ and ‘non-seizure’ classes from intracranial EEG recordings, detection rate, with 100% accuracy rate. Further, Lahmiri et al. [43] exploited GHE with SVM, to classify the ‘seizure’ and ‘non-seizure’, and also they found 100% accuracy in less time. Here, the good indication is that authors claim the good accuracy in less time for seizure detection. But, the authors did not clearly define how many times the seizure can be detected. In another study by Al Ghayab et al. [88], the obtained accuracy is 100% as a result of using the concept of Information gain theory, to extract and rank the meaningful features from EEG signal dataset. The least square-support vector machine (LS-SVM) is then applied to classify the seizure cases. Moreover, due to the ‘black-box’’s nature of applied classifiers, the authors could not explore any other related aspects in terms of Knowledge discovery. Zabihi et al. [81] did patient-specific seizure detection using SVM classifier on the processed dataset with a good set of features, comprising time-domain, frequency-domain, time–frequency domain, and non-linear feature. The performance of their model has achieved an average of 93.78% sensitivity and a specificity of 99.05%. Here, it is noteworthy that they skip an important feature—‘line length’, from the available literature, which is prominently used in seizure detection. We also argue that CHB-MIT dataset [60] is imbalanced because, in an hour(s) of recording, a seizure time span is for a few seconds.

*Observations*

The main issue with ‘black-box’ classifiers is that they only make prediction without providing logic rules or patterns. That is why, they are not recommended for extracting sensible knowledge. For example, for class imbalance issues in EEG datasets, insufficient related literature is found, and the researchers who attempted to work on this problem did not provide a conceivable solution as to how to solve the class imbalance issue whilst detecting the seizure.

### Seizure detection based on non-black-box classifiers

‘Black-box’ classifiers are unable to express their classification procedure for human interpretation [67, 104, 116]. Consequently, there are fewer chances for knowledge discovery and better accuracy performance. Therefore, the concept of ‘non-black-box’ classifiers such as decision trees, and decision forests came into practice.

Chen et al. [119] first introduced the decision tree to the EEG dataset for seizure detection. Kemal and Saleh [120] used a C5.0 decision tree [121] algorithm to explore the logic rules for seizure detection, with an average accuracy of 75%. When the same C5.0 was applied to the same dataset processed by Fourier transformation the obtained accuracy with cross-validation was, however, 98.62%. A few related works are been available, where only a decision tree method is applied seizure detection because of less accuracy and a *limited number of patterns* obtained from the logic rules of a decision tree [122]. As a result, both the knowledge discovery and accuracy suffer. However, this gap can be filled by applying decision forest approaches instead [51, 57, 123].

Through the literature, it is found that the decision forest approaches are more effective than the single decision tree [57, 124], because the decision tree often gives a confined set of rules and overfitting issue is also raised [68]. The rules are extracted from training data by a decision tree that generates either limited or a single set of logic rules (Say, wherever *C2_Entropy* value $\le 101.01$ then $Class\_value=seizure$) and stops growing the tree further records in the training dataset once the rule is accepted. However, if we generate a decision forest on the training data, we can achieve multiple sets of decision trees with the combination of sensible logic rules and a higher accuracy rate due to the majority voting method [57]. Decision forest classifiers [54, 68] are the type of ensemble methods that are used frequently. These are also used in seizure detection as they provide a high accuracy rate which depends on the majority voting method from the ensemble of decision trees. Moreover, they produce more logic rules as multiple decision trees from the training data (*D*) [123]. These logic rules are humanly interpretable, and data scientists can easily interrelate them with other seizure-related information from EEG datasets.

Siddiqui and Islam [125] used Systematic Forest (SySFor) to detect the seizure on ECoG without epoch reduction. Further, Siddiqui et al. [63] applied two decision forests—Systematic Forest (SysFor) [123] and Forest CERN [51] on nine statistical features for quick seizure detection using the concept of epoch length reduction. It is based on dividing the size of training dataset *D* into $D_1, D_2$, ...$D_n$ and testing the accuracy at every epoch of the dataset. These sub-datasets are in descending order in terms of time duration. If the seizure can be detected in a shorter epoch length without a decline in accuracy, then we can use the same one, which results in fast seizure detection. They achieved 100% accuracy. The limitation of this work is that authors have taken the dataset of a single patient, this could be tested for more patients. Several researchers have taken the advantages of random forest classifier for detecting the seizures [76, 78, 82, 126]. Because researchers/data scientists are able to see the logic rules and interpret them correspondingly. Moreover, it also provides good accuracy [44, 76–78, 80, 82]. Donos et al. [44] applied decision forest classifier—random forest, on time and frequency domains’ feature, which was extracted from an IEEG (Intra-cranial EEG) dataset. It helped in selecting the intra-cranial channels for early seizure detection in a closed-loop circuit. The results claimed that the system can detect the seizure with 93.8% sensitivity. Wang et al. [94] developed the greedy approach of random forest, i.e., forest-grid search optimization (RF-GSO), with this method and they found 96.7% accuracy. The shortcoming of this technique is that the performance could decline if EEG signals are too noisy. Tzimourta et al. [93] applied random forest to monitor seizure activities on the two benchmark epilepsy datasets [64, 65], the reported performance is 99.74%. Pinto-Orellana and Fábio R. Cerqueira [76] also used the random forest on the processed CHB-MIT dataset by a Spectro-temporal feature, and 70s, and the accuracy of each block is 98.30%.

Truong ND et al. [82] had carried out novel work of channel selection whilst detecting the seizure. Their key contribution is that they also focus on channels contributing mostly to automatic seizure detection. They used the random forest to solve channel selection and seizure detection, and which achieving 96.94% area under the curve (AUC). In another work, Mursalin et al. [80] proposed a method for seizure detection by selecting features with an Improved Correlation-based Feature Selection(ICFS). Basically it is a fusion of time and frequency domain. Then, a random forest classifier was applied for the seizure detection model. The obtained average classification accuracy by this approach was 98.75%.

Some other works have used an ensemble of ‘non-black-box’ classifiers such as boosting, bagging and random subspace [78, 127]. Yan et al. [78] applied a boosting classifier achieving 94.26% of accuracy, although the results were not as impressive as the ones obtained by [44], which used a random forest classifier. Hosseini [128] used Random subspace classifier along with an SVM classifier, to classify and detect seizures. Here, the benefit of applying a subspace on big datasets is to divide them into sub-datasets based on the random subspace concept, and then the SVM classifier was applied to each sub-dataset. Ensemble accuracy (EA) was calculated by the majority voting method, which was 95%. Apart from this study, the same authors of Hosseini et al. [126] recently did another research using an ensemble of classifiers. First, they created bootstrap samples using a random subspace method, and then applied classifiers such as SVM, KNN, extended nearest neighbor (ENN), and multilayer perceptron (MLP) obtaining 97% accuracy. Hussein et al. [100], proposed a novel feature extraction method, i.e., L1-penalized robust regression (L1PRR), which uses three common symptoms during seizures—muscles artifacts, eyes movement, and white noise. Inputting these features help the random forest classifier to obtain 100% accuracy.

*Observations* In comparison to decision trees, decision forest classifiers are tremendously used on brain datasets for exploring different research goals. It is difficult to suggest a particular classifier whilst dealing with a high-dimensional dataset, but a random forest classifier can be a capable classifier. However, it also criticizes that not all the ‘non-black-box’ classifiers are peculiar to detect seizures and have also pointed out the objection on the drawback of using a single decision tree classifier.

### Seizure detection based on black-box and non-black-box machine learning classifiers

From the literature, it is found that just a single machine learning classifier is not sufficient. Therefore, to take advantage of both ‘black-box’ and ‘non-black-box’ classifiers, some researchers utilized them in their experiments. This section provides a comprehensive review of classifiers applied together to detect the seizure.

Acharya et al. [111] used the ensemble of seven different classifiers—Fuzzy surgeon classifier (FSC), SVM, KNN, Probabilistic neural network, GMM, decision tree and Naïve Bayes for distinguishing the three states of a patient as ‘normal, ‘pre-ictal’ and ‘ictal’. The overall accuracy is 98.1%. Fergus et al. [83] also used distinct classifiers such as linear discriminant analysis (LDA), quadratic discriminant classifier (QDC), logistic classifier, uncorrelated normal density-based classifier (UDC), polynomial classifier, KNN, PARZEN, SVM, and decision tree on the processed data with seven features such as entropy, RMS, skewness, and variance. They contributed that the detected patient is suffering from a ‘Generalize seizure’ (means affecting whole brain region) across different patients without prior information about the seizure focal points. Mursalin et al. [101] proposed a method to reduce the data size, statistical sampling technique called optimum sample allocation technique, and to reduce the features they develop a feature selection algorithm. The analysis was done on the combination of five classifiers—SVM, KNN, NB, Logistic Model Trees (LMT) and Random forest.

Rand and Sriram [95] used four classifiers such as SVM, KNN, random forest, and Adaboost on a high-dimensional dataset prepared by 28 features. Their result shows that SVM outperforms on the cubic kernel. In another study, Manzouri et al. [98] used SVM and random forest on the dataset produced by 10-time and frequency features. In comparison to SVM-based detector, random forest classifier outperforms. Subasi et al. [96] achieved 100% of accuracy using four machine learning classifiers such as ANN, KNN, SVM, and random forest on two popular datasets—Freiburg and CHB-MIT to classify the three different states of seizures ‘pre-ictal’, ‘ictal’, and ‘inter-ictal’. Sharma et al. [102] proposed an automated system using iterative filtering and random forest for classifying the EEG signals. This work achieved classification accuracies of 99.5% on BONN dataset (A-E), for A versus E subsets, 96% for D versus E subsets, and 98.4% for ABCD versus E classes of EEG signals. Birjandtalab et al. [77] used two classifiers for different purposes; KNN is used to discriminate the ‘seizure’ and ‘non-seizure’ classes, whereas random forest is used to explore the significant channels. Here, the random forest also helps in the dimension reduction problem. The main benefit of selecting suitable channels is that it helps in providing relevant required information from the chosen channels, and reduces the computational cost of a classifier too. However, the authors did not mention here the important information from channel selection like finding the seizure location from the brain scalp. The main critic in [95, 98, 101] is that because of a large number of features, the attribute size of dataset will increases, and as a result the accuracy and computation time suffer.

#### Observations

We observe that some work used an ensemble of distinguished classifiers to take the benefits separately. For example, influential channel selection can be independently done using decision forest classifiers like a random forest. But authors used other classifiers such as SVM and KNN for classifying the seizure records with good accuracy.

## Seizure localization

After a successful seizure detection, localization is an essential task for epileptic surgery [129–131]. Typically, localized seizures can be cured by surgery which arises either from the left or right region of the brain. The seizure monitoring tools such as ECoG and EEG are prominently helpful to identify the seizure location. The electrodes/channels are implanted in a non-invasive (for EEG) and an invasive manner (for ECoG). Their positioning is based on the 10/20 (10–20) International system, which helps in identifying the seizure location [132]. The concept of seizure localization means identifying the region of the brain affected by a seizure. Though some types of seizures such as ‘tonic-clonic’ are cured by anti-epileptic drugs (AED), patients with partial seizures in some cases might go for surgery [13]. To solve this problem, finding the seizure location is an essential and challenging task for neurologists and neurosurgeon [129, 130]. The surgical target is to find a point/location/focal area from where a seizure is originating. The 10–20 positioning system gives some clues for identifying the location of a seizure. Recently, computational and machine learning methods have been applied to identify a seizure location [130, 133].

Acar et al. [133] used trucker and non-linear multi-way Trucker kernels, and claimed that other classifiers such as SVD and principal component analysis (PCA) were unable to localize a seizure. Ghannad-Rezaie [134] applied an advanced swarm intelligence algorithm to seizure data for finding seizure location. Their study produced some appreciable results, and explored whether the patient’s temporal lobe was affected by a seizure or not. They also suggested that SVM might be able to detect the seizure location. Moreover, they also focused on the reduction of ECoG electrodes. Mansouri et al. [135] proposed an algorithm for Seizure localization, which was tested on 10 sec of EEG dataset from Karuniya University. Here, they have taken the small-size dataset, because recording usually takes several hours. If they had tested on a big dataset, it would have been much better. Fakhraei et al. [130] calculated the sensitivity of each region of the brain. The confident prediction rate (CPR) was compared with the AUC of ROC plots obtained by six classifiers from the dataset of 79 patients (31 males, 48 females) with 197 medical features. The study found that CPR was more suitable than ROC. They also explored that 43 patients had the temporal lobe epilepsy (TLE) on their left sides whilst 36 patients had it on the right sides of their brains. Likewise, Rai et al. [136] proposed a method for identifying the focal points of the seizure by applying two entropy-based features—‘renyi entropy’ and ‘negentropy’ with the neural network classifier. Siddiqui et al. [63] localize the seizure using two decision forest classifiers, and their results showed that the left hemisphere of a brain was more affected by the seizures.

*Observation*

It is found that compared to seizure detection, machine learning classifiers have not been extensively applied for seizure localization. But some literature exist on this problem. In these reported works, authors did not mention the percentage of the affected region of the brain by a seizure, and they were not able to identify the exact location at the lobes such as occipital, frontal, parietal left and parietal right. Although, it is not our primary objective in this review paper, whilst discussing the related published research, we found some interesting clues for seizure localization.

## Problems identified in existing literature

One of the most significant and decisive steps is to select suitable statistical features because each channel or electrode implanted on the brain provides different statistical measures. Undoubtedly, earlier researchers made their consistent efforts to find the best features. Whilst some researchers used many features [34, 79], the others applied a few features [31, 36, 108, 112, 137] for detecting the seizure. As a data scientist, it is very important to see the different statistical perspectives of each brain signal by analyzing the statistical properties of the features such as entropy, energy, and skewness. And we must not focus on taking irrelevant feature(s) as such since it will unnecessarily increase the dataset size. Consequently, it will be more a burden to machine learning classifiers than a benefit, and if we take few features as previous researchers did [71, 73, 79], this will give the low-dimensional dataset and it will not be beneficial for an effective knowledge discovery process. Therefore, we should select those potential features that can to provide logical results. Hence, it is advisable to select a group of features to avoid a burden to the machine learning classifiers and to get help in related knowledge discovery.

Each classifier has its own merits and demerits, depending on the dataset attributes and requirements [138]. In general, it is very difficult to point out which classifier was the most effective for brain datasets. To identify the capable classifier, several classifiers have been tested on EEG datasets and their performance has been evaluated, and the one which performs well is to be considered in solving seizure detection and imparting knowledge discovery. The literature reveals that previous researchers had applied different approaches, most of which were from ‘black-box’ such as ANN, KNN and SVM. The biggest shortcoming in them is that they are unable to provide the appropriate explanations for patterns and the logic rules hidden inside the models. That is why, they are not suggested for remarkable knowledge discovery process. Data scientists may not explore the internal processing of patterns [51, 104]. However, from the literature, it is noted that the ‘non-black-box’ approach, especially, random forest, is widely used for seizure detection [44, 76, 77], because of its nature of generating bootstrap samples [124, 139] whilst building a decision forest. An analysis has been done to estimate the performance of machine learning classifiers on EEG datasets and has been found that ensemble non-black-classifiers performs effectively [104]. We argue that the random forest is based on bootstrap samples and it misses some influential attributes, because it randomly selects the attribute and sometimes generates the same set of logic rules also. As a result, sometimes, it creates irrelevant information too. To overcome this issue, we also suggest some other decision forest algorithms such as SysFor [123] and Forest CERN [51] methods in seizure detection.

All these findings on seizure detection raise few interesting research questions such as selecting suitable statistical features and machine learning classifiers to take less computation time as dataset has a high volume with high dimension, and the most significant missing information from machine learning classifiers is locating the accurate point of seizure at the brain lobe(s).

### Class imbalance issue in seizure detection

Class imbalance is one of the serious problems [140] in machine learning and the majority is seen in medical datasets [141], particularly in EEG signals. This is because the duration of EEG recording is long, time-consuming and seizure duration is for a few seconds, which results in being prone to errors [91]. As a result, the dataset becomes highly imbalanced. Previous researchers have focused on seizure detection. Over the last few years, researchers have been focusing on the class imbalance challenge whilst detecting the seizures, and attempting to solve it by applying different conventional approaches with some novelties. Javad Birjandtalab et al. [91] used ANN with a weighted cost function to imbalanced EEG dataset, by achieving 86% F-measure. El Saadi et al. [142] obtained 97.3% accuracy using the under-sampling method with the SVM classifier. In another work by Saadullah and Awais [143], they used a combination of SMOTE and RUSTBOST techniques for detecting seizure to imbalance seizure data with 97% accuracy. However, the research done by Yuan Qi et al. [86] was very close to the satisfactory result as they assigned the heavy weights to a minority class of the data to maintain the effective balance and solved the biasing issue. The main critique of this work is that the authors did not mentioned what weights were assigned and what was their threshold level? Here, we argue that despite of EEG data are highly imbalanced as a result of their long-hour EEG recordings, the recordings continue until the seizure is detected. The seizure(s) time spans from only seconds to minute(s). Although researchers [76, 86, 117, 143] made their efforts in addressing this issue using both ‘black-box’ and ‘non-black-box’ classifiers, they did not propose any justifiable solutions, in terms of how big weights should be assigned to the minority (seizure) classes.

## Overall observation about capable classifiers and statistical features

It is challenging to suggest that a specific classifier should be capable for seizure detection. If we discuss classifiers, three constraints are very important whilst selecting a classifier—able to handle the high-dimensional dataset, high accuracy of the model, and able to retrieve the sensible knowledge. Not all machine learning classifiers are suitable for seizure detection and knowledge discovery tasks, mainly because of their *black-box* nature. This means that the logic rules/patterns are not visible and understandable to data scientists. In ‘non-black-box’ classifiers amongst decision trees [53] and decision forests [54], only decision forest algorithms are more capable, because the logic rules and knowledge discovered by a single decision tree are often limited and insufficient. For example, if we build a decision tree on a training dataset—it provides a limited or single set of logic rules and stops growing the tree further as all the data points in the training set accept that rule. On the other hand, if we build a decision forest on the same training set, we get multiple decision trees with more sensible logic rules. Siddiqui et al. [104] have done the analysis on CHB-MIT dataset to know which classifier performs better. For this, they applied two black-box (SVM and KNN) and two non-black-box (decision tree and ensemble of trees i.e., bagging, random subspace, boosting); they found non-black box classifier (ensemble) outperforms compared to other classifiers of black-box. Even ensemble also performs better than a single decision tree which is a non-black box classifier. Siddiqui et al. [63] applied two decision forests—Systematic Forest (SysFor) and Forest CERN for quick seizure detection using the concept of epoch length reduction. They achieved 100% of accuracy. Similarly, Hussein et al. [100] also achieved 100% accuracy using decision forest–random forest approach.

The literature reveals that in the last few years, ‘non-black-box’ classifiers, particularly decision forest approach, were widely used on brain datasets of EEG and ECoG for different research goals [76, 82, 94, 144]. The reasons for using the *decision forest* for seizure detection are as follows:

1. A decision forest overcomes some of the disadvantages of a decision tree. A decision tree discovers only a single set of logic rules from an input dataset. The logic rules that are discovered by a single decision tree may fail to correctly predict and classify the class values;
2. A decision forest can produce more set of logic rules/patterns compared to a single decision tree and there is a high chance of good prediction/classification compared to a single decision tree;
3. Able to handle high-dimensional sets;
4. Due to its ensemble nature a decision forest mostly produces a high accuracy compared to a single tree and other classifiers [54];
5. Less computational time (specifically for Random forest);
6. Logic rules are clear and humanly interpretable such as analysts/domain experts can easily understand and suggest best opinions. For example, affected brain lobe by seizure, identifying suitable statistical features, etc.

Furthermore, many statistical features have been used for seizure detection. However, a comparison between them is difficult because of their heterogeneous nature. Some researchers used a single feature such as energy and entropy. On the other hand, a combination of statistical features such as energy, kurtosis, line length, entropy, skewness, max, standard deviation, and min may produce promising outcomes. Most research [34, 46, 92, 100, 109, 145] have achieved better results using these features. The novelty of [29, 63, 104, 125] is the selected nine statistical features are able to assist in seizure detection with high accuracy, i.e., 100%. This also provides the clue about seizure localization with the help of sensible logical rules. Hence, the selected group of features will not be a burden to the machine learning classifier but it will assist in related knowledge discovery.

## Research directions in seizure detection

In this research analysis, we surveyed different machine learning classifiers used for seizure detection. No doubt, the progress of the persistent attempt has been found in this topic but few interesting research questions are also raised. In this section, we identify significant challenges which can uplift the future research in this area.

1. Selecting suitable statistical features and machine learning classifiers to take less computation time as the dataset has a high volume with a high dimension.
2. Accurate seizure detection on imbalanced datasets of long duration EEG recording datasets.
3. Quick seizure detection on long-hour EEG recording.
4. Whilst selecting the machine classifier it should be kept in mind that the classifier does not miss any necessary EEG channel/electrode.
5. Knowledge discovery from machine learning classifiers such as seizure localization which exactly points affected brain lobe(s), channel importance, and based on participating channels in seizure a knowledge could be provided to neurologist or neurosurgeon for suggesting epilepsy category.

## Conclusion

With the increase of epilepsy, its accurate detection becomes increasingly important. A major challenge is to detect seizures correctly from a large volume of data. Due to the complexity of EEG signals in such datasets, machine learning classifiers are suitable for accurate seizure detection. Selecting suitable classifiers and features are, however, crucial.

As such, this paper has comprehensively reviewed machine learning approaches for seizure detection. As a result, we conclude that ‘non-black-box’ classifiers—decision forest (ensemble of decision trees)—is most effective. This is because it can produce multiple sensible, explanatory logic rules with high accuracy of prediction. Further, it can help discover some relevant information such as seizure localization and exploring seizure types. On the contrary, ‘black-box’ classifiers cannot generate logic rules, although they can achieve high predictive accuracy. As for selecting suitable features, we should select those that can provide logical results. By the review of the literature, the use of the features such as entropy, line length, energy, skewness, kurtosis, and standard deviation can achieve 100% accuracy in the classifiers. We suggest not to use the irrelevant features as the dimension of the data increases. This is because the computation cost of a classifier will grow high, and it may also produce insensible patterns. If we use just one or two features such as line length and energy, the low-dimensional dataset will be generated. However, this dataset will not be fruitful for the knowledge discovery process.

This review paper has provided new perspectives to data scientists who are working on epileptic seizure detection using EEG signals. In summary, this paper focuses on the review of selecting machine learning classifiers and suitable features.

## Data Availability

Not applicable.
